# Do Natural Proteins Differ from Random Sequences Polypeptides? Natural vs. Random Proteins Classification Using an Evolutionary Neural Network

**DOI:** 10.1371/journal.pone.0036634

**Published:** 2012-05-16

**Authors:** Davide De Lucrezia, Debora Slanzi, Irene Poli, Fabio Polticelli, Giovanni Minervini

**Affiliations:** 1 European Centre for Living Technology, University Ca’ Foscari Venice. Venice, Italy; 2 Dept. of Environmental Sciences, Informatics and Statistics, University Ca’ Foscari Venice, Venice, Italy; 3 Dept. of Biology, University of Roma Tre. Rome, Italy; 4 National Institute for Nuclear Physics, Roma Tre Section. Rome, Italy; Universitat Pompeu Fabra, Spain

## Abstract

Are extant proteins the exquisite result of natural selection or are they random sequences slightly edited by evolution? This question has puzzled biochemists for long time and several groups have addressed this issue comparing natural protein sequences to completely random ones coming to contradicting conclusions. Previous works in literature focused on the analysis of primary structure in an attempt to identify possible signature of evolutionary editing. Conversely, in this work we compare a set of 762 natural proteins with an average length of 70 amino acids and an equal number of completely random ones of comparable length on the basis of their structural features. We use an *ad hoc* Evolutionary Neural Network Algorithm (ENNA) in order to assess whether and to what extent natural proteins are edited from random polypeptides employing 11 different structure-related variables (*i.e.* net charge, volume, surface area, coil, alpha helix, beta sheet, percentage of coil, percentage of alpha helix, percentage of beta sheet, percentage of secondary structure and surface hydrophobicity). The ENNA algorithm is capable to correctly distinguish natural proteins from random ones with an accuracy of 94.36%. Furthermore, we study the structural features of 32 random polypeptides misclassified as natural ones to unveil any structural similarity to natural proteins. Results show that random proteins misclassified by the ENNA algorithm exhibit a significant fold similarity to portions or subdomains of extant proteins at atomic resolution. Altogether, our results suggest that natural proteins are significantly edited from random polypeptides and evolutionary editing can be readily detected analyzing structural features. Furthermore, we also show that the ENNA, employing simple structural descriptors, can predict whether a protein chain is natural or random.

## Introduction

The question whether extant proteins are the exquisite result of natural selection or rather they represent random co-polymers slightly edited by evolution has stirred an intense discussion for the last twenty years for its implications in origin of Life [1], macromolecule aetiology [2], [3] and evolution at large [3]–[5].

From the molecular point of view, protein evolution can be viewed as a search and optimization process in the sequence space to identify suitable sequences capable to fulfill a functional requirement. In addition, any biological requirement (i.e. catalysis, binding, structure) must be viewed as a multi-objective problem so that any functional protein is a trade-off solution to different problems such as function, solubility, stability and cellular environment (i.e. interaction with other proteins). Thus, extant proteins can be considered as a highly specific output of a long and intricate evolutionary history and accordingly they are as unique as the evolutionary pathway that produced them.

This perspective has been challenged by several authors who raised the problem of whether and to what extent proteins are the unique product of evolution or a sheer accident [4]. The rational beyond this argument relies on the vastness of the sequence space which grows exponentially with the length of the protein. This space is so astronomically big that an exhaustive search and optimization is impossible [5], [6] and therefore some randomness seems inevitable during the evolutionary process. Furthermore, some authors put forward the notion that extant proteins are the mere output of a contingent process dictated by the simultaneous interplay of several independent causes so that extant proteins can be regarded as simply a frozen accident [1].

Ptitsyn was the first to argue against the common tenet that proteins are the result of a directed selection in the course of biological evolution. In his work he suggested that typical three-dimensional structures of globular proteins are intrinsic features of random sequences of amino acid residues. Therefore, Ptitsyn concluded that primary structures of proteins are “mainly examples of random amino acid sequences slightly edited in the course of biological evolution to impart them some additional (functional) meaning” [7]–[9]. This hypothesis was corroborated by Weiss and Herzel who investigated possible correlation functions in large sets of non-homologous protein sequences. They found that correlation in protein primary sequences are weak and do not significantly differ from those found in random surrogates [10]. In a later work the two authors studied the complexity of large sets of non-redundant proteins and a dataset of randomly generated surrogates by a number of different estimators to measure the Shannon entropy and the algorithmic complexity. Their results show that proteins are fairly close to random sequences, indeed natural proteins have approximately 99% of complexity of random surrogates with the same amino acids composition. These results support the idea that protein primary sequences can be regarded as slightly edited random strings [11]. The same general conclusions were drawn by other authors who approached the same problem from a different prospective. Crooks and Brenner attempted to unveil correlation between protein secondary structure and amino acids content in primary sequences. Results supported the conclusion that correlations at primary sequence level were essentially uninformative and that the protein sequence information content could be effectively explained assuming a random model of protein generation [12]. Lavelle and Pearson investigated whether folding constraints and secondary structure preferences significantly bias amino acid composition and usage in proteins. Authors compared the frequencies of four- and five-amino acid stretches in a non-redundant proteins dataset to the frequencies expected for random sequences generated with four independent models. Their results showed that amino acid stretches do not appear to be significantly biased, indeed primary sequences appear to be “under very few constrains, for most part, they appear random” [13].

These results support the conclusion that primary structures of extant proteins are basically random amino acid sequences which have only been “edited” and “refined” during biological evolution in order to acquire stability and function.

In despite of these results, other authors came exactly to the opposite conclusion. Panke and co-workers attempted to highlight subtle deviations of extant protein sequences from pure randomness by mapping protein sequences onto a one-dimensional space by decoding proteins primary sequences using chemico-physical descriptors such as Coulomb interaction, hydrophobic/hydrophilic interaction and hydrogen bonding [6]. Using these three different descriptors, authors found pronounced deviations from pure randomness. Authors reasoned that these deviations are evidence for a physically driven stage of evolution. In particular, authors advocate that these deviations seem directed toward minimization of the energy-frustration of the three-dimensional structure which witnesses a clear evolutionary fingerprint.

Munteanu and co-workers [14] used a Randic’s star network to convert protein primary structure into topological indices which describe a real protein as a network of amino acids (nodes) connected by peptide bonds (arches). Authors compared two sets of proteins: a set of 1046 natural protein chains derived from the CulledPDB [15] and a second dataset with the same size of random amino acid sequences. Authors developed for the first time a simple classification model based on statistical linear methodologies capable to effectively classify natural/random proteins with a remarkable predictive ability of 90.77%. Thus, the works by Pande and Munteanu suggest that extant proteins are indeed significantly different from random co-polymers and natural sequences do display a clear evolutionary signatures.

By and large there is a robust body of literature specifically addressing the question of whether extant proteins are significantly edited from random polypeptides or rather they “represent memorized random sequences”, however these works come to contradicting conclusions and fail to provide a conclusive answer. Despite the different findings, all these works share a common feature: they attempt to tackle the question by investigating proteins primary sequences.

Conversely, in this work we extend and refine a previous study [16] by comparing a set of 762 natural proteins with an average length of 70 amino acids and an equal number of completely random ones of comparable length on the basis of their structural features. The rationale beyond is that, in the vast majority of cases, proteins exert their physiological functions by virtue of their 3D shape, thus any possible signature of evolutionary editing should be searched at the level of the tertiary structure rather than at the level of the primary one. Toward this goal, we employed 11 different structure-related variables to develop an Evolutionary Neural Network Algorithm (ENNA) capable to correctly distinguish natural proteins from random ones with an accuracy of 94.36%. Besides, the analysis of the structural and functional features of some random polypeptides misclassified by the ENNA algorithm as natural ones revealed a significant structural homology to extant proteins.

All together, our results suggest that natural proteins are significantly edited from random polypeptides and evolutionary editing can be readily detected analyzing structural features. Furthermore, we also show that the Evolutionary Neural Network Algorithm employing simple structural descriptors can predict whether a protein chain is natural or random.

## Results

We initially investigated a set of 902 natural proteins (Nat) whose tertiary structure was experimentally resolved (either by NMR or X-ray) and a set of 20494 completely random protein (Rnd) sequences generated using a uniform amino acid frequency distribution with no significant homology to natural ones. The Nat dataset was derived from the Protein Data Bank [17] and composed of natural proteins with experimentally resolved 3D-structure and an average length of 70 amino acids (within a range of 55 to 95 amino acids) comparable to the length of Rnd (70 amino acids long sequences). The dataset was cleaned up to eliminate protein fragments and proteins involved in the ribosomal complex. The analysis of the Nat dataset showed that there is a comprehensive representation of proteins fold types, even though proteins with extended beta-sheet are under-represented due to length constraints.

Eleven different structure-related variables were calculated for both data sets: net charge, volume, surface area, coil, alpha helix, beta sheet, percentage of coil, percentage of alpha helix, percentage of beta sheet, percentage of secondary structure and surface hydrophobicity. The structure-related variables were calculated directly from the PDB file for the Nat dataset, whereas the same variables were computed from tertiary structure models for the Rnd dataset.

First, we performed a pre-processing of the data to remove the outliers that could affect subsequent analysis. Outliers were identified as those proteins with one or more structure-related variables markedly deviating from the average. In our case, we considered as outlier any protein with one or more structure-related variables falling in the tail of estimated probability distribution (i.e. p<0.005 and p>0.995). In our sample, we detected 140 natural proteins and 2029 random proteins with one or more structure-related variables markedly deviating from the estimated average. These proteins were removed reducing the number of the observations to 18465 for the set of random proteins and to 762 for the set of natural proteins. The two dataset were considerably different in size, with random proteins largely outnumbering the natural ones; thus in order to avoid any possible bias we performed the analyses using a random sample of observations drawn from the random proteins equal to the size of the Nat dataset (*i.e.* 762).

**Figure 1:**
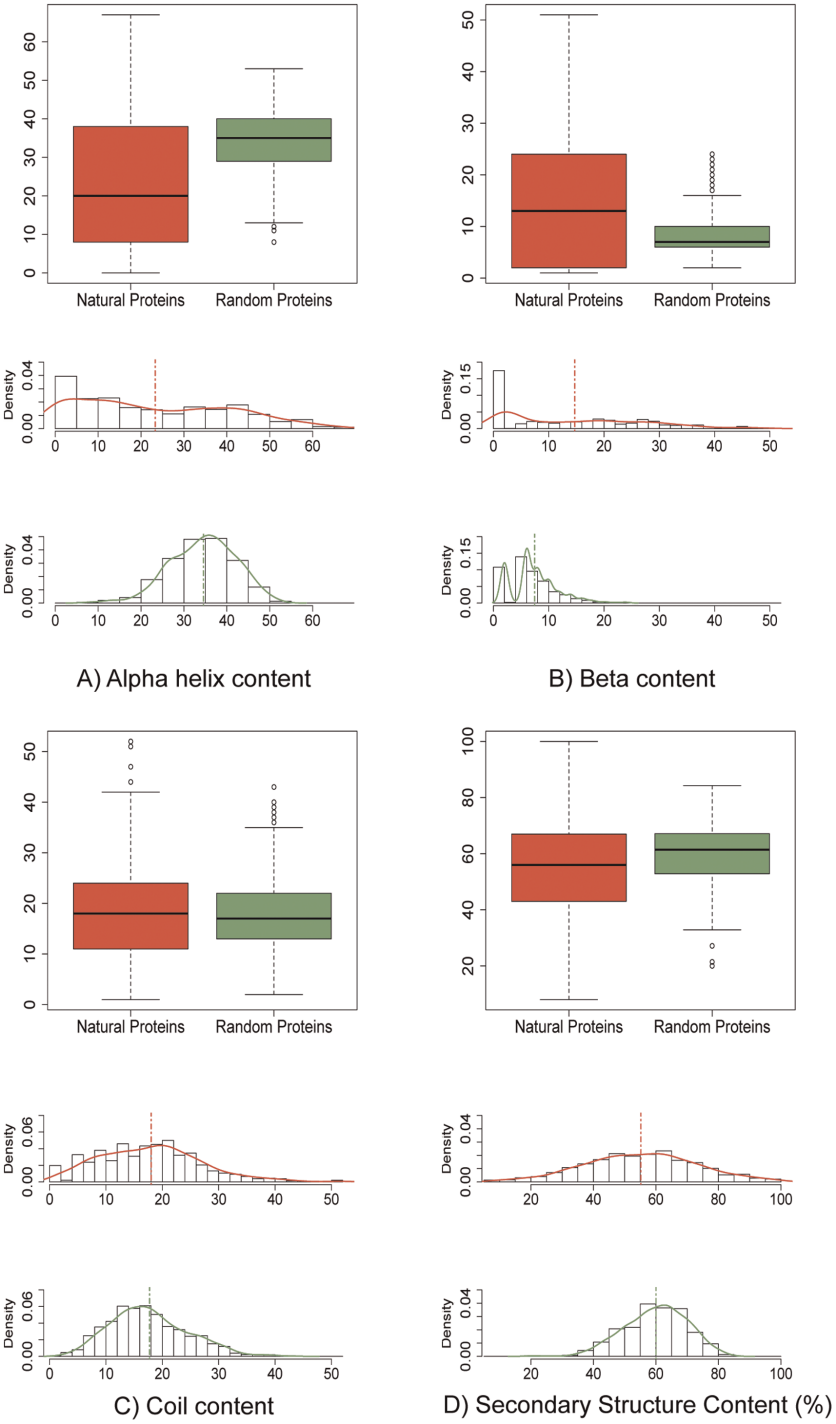
Descriptive statistics of the structural properties of random and natural proteins. Descriptive comparison of natural and random proteins by means of: boxplot of the variables distribution in the two classes (top) and histogram of the variables distribution with the corresponding Kernel density estimate (bottom).

**Figure 2:**
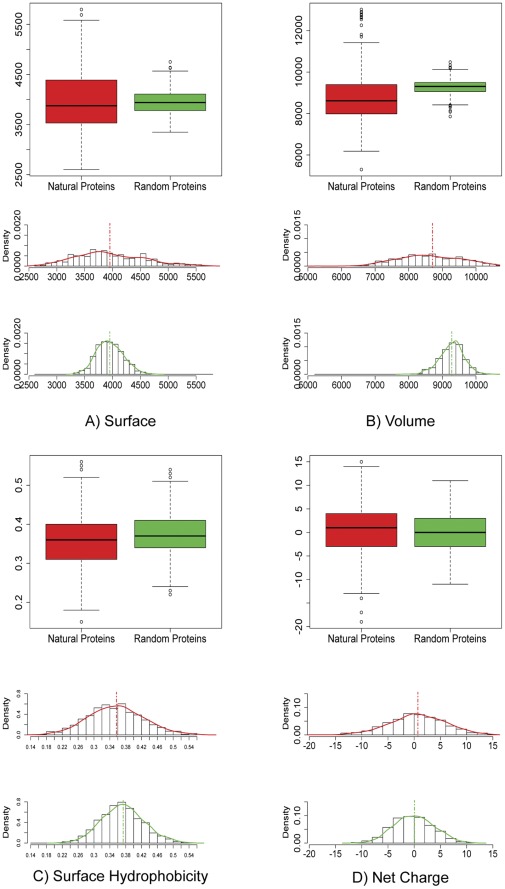
Descriptive statistics of the structural properties of random and natural proteins. Descriptive comparison of natural and random proteins by means of: boxplot of the variables distribution in the two classes (top) and histogram of the variables distribution with the corresponding Kernel density estimate (bottom).

**Figure 3:**
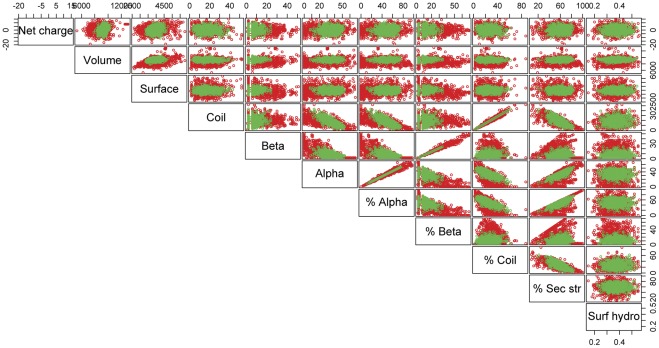
Scatter plot of the structural properties. Overlap of the scatterplots for the two classes of proteins for each variable pairs. Random proteins are represented in green, Natural ones are represented in red.

A first exploratory data analysis was carried out to assess whether there were any significant difference in the structure-related variables observed in the two data-sets. First, we performed a Gaussian distribution test for every individual variable which led to reject the hypothesis of Gaussianity with a test significance level of 0.01 for all variables except for percentage of secondary structure and surface hydrophobicity for the natural dataset and surface hydrophobicity and surface area for the random protein dataset. For all variables we derived measures of location, index of dispersions, correlations matrix, in addition boxplots and scatter plots were built to compare the two data sets. Statistical analyses highlighted that both mean and variance were significantly different for all variables with a test significance level of 0.01 except for variables coil, percentage coil and surface area (Table 1). The first striking outcome is that in general natural proteins show a broader distribution with respect to random ones for most of the variables investigated (Figure 1 and 2). This general feature can be explained considering that random proteins represent statistical copolymers and therefore their structural features are centered around the mean with a variance equal to the one expected by the correspondent probability density function. Conversely, natural proteins structure-related variables significantly depart from expected values due to the tuning effect of natural selection. We computed scatter plots for the two classes of proteins for each variables pair (Figure 3). The scatter plots’ centroids generally overlap for the two datasets. Conversely, their distributions in the 2D plot are remarkably different, with natural proteins more broadly dispersed. This observation supports the idea that natural evolution has extensively refined proteins’ structural and chemo-physical properties to meet functional requirements.

**Table 1:** Average values of the structure-related parameters.

Variable Name	Mean			Standard deviation		
	Natural	Artificial	p-value^1^	Natural	Artificial	p-value^2^
Net charge	0,7100	0,0591	0,0023	5,335634	3,753989	≈ 0
Volume	8706,4310	9279,6090	≈ 0	1067,166	356,8877	≈ 0
Surface	3952,0580	3951,5030	0,0383	603,5493	237,2047	≈ 0
Coil	18,0276	17,7349	0,7493	9,03813	6,887919	≈ 0
Beta	14,7192	7,4567	≈ 0	12,33831	4,236918	≈ 0
Alpha	23,3032	34,5538	≈ 0	17,71133	7,646621	≈ 0
% Alpha	33,8530	49,3625	≈ 0	25,9784	10,92395	≈ 0
% Beta	21,3976	10,6532	≈ 0	17,86945	6,052317	≈ 0
% Coil	26,0394	25,3356	0,3870	12,77989	9,839893	≈ 0
% Secondary structure	55,2100	60,0151	≈ 0	17,92994	10,10049	≈ 0
Surface hydrophobicity	0,3568	0,3738	≈ 0	0,0695038	0,05349438	≈ 0

1 evaluated by Wilcoxon test.

2 evaluated by Fligner-Killeen test.

Average values of the structure-related parameters were calculated for natural proteins and random ones. Both mean and variance were significantly different for all variables with a test significance level of 0.01 except for variables Coil, % Coil and surface area.

The significant differences of the structural features between the two datasets prompted us to develop a classification method capable of distinguish the natural proteins from random ones. In this work we employed a Evolutionary-based Neural Network classification Algorithm referred as ENNA [18], which evolves populations of neural networks where the inputs are the structure-related variables and the output is the class of the protein (Nat or Rnd). Briefly, ENNA generates a first random population of networks with the topology of a 2-hidden layers neural networks. This population is formally described as a set of sequences with dichotomic variables (each sequence is a vector of zeros - ones values) representing the input of each network. Each element of the sequence describes the presence or the absence of a particular structure-related variable. The topology of these networks, involving different variable compositions, was selected in a random way (first generation of networks), and the response of each network was derived with a two classes structure: natural and random proteins. The process then builds a genetic algorithm to evolve the population of networks in a number of generations to identify a precise classification rule. We evaluated the response of each network deriving a net misclassification rate by 10-fold cross validation procedure: the sequences with smaller values are identified as the more promising solutions. Then we applied to the network population the classical genetic operators, such as natural selection, crossover and mutation, in order to achieve the next generation of promising sequences. At the end of the evolutionary process we achieved the population of Neural Networks with the smaller misclassification rates. The analysis of the last population of Neural Networks revealed that only a limited number of structure-related variables were required to correctly classify the two dataset, namely: Volume, Coil, Alpha, and Surface hydrophobicity. These variables had a probability close to 1 to occur in the last population, thus they can be considered robust in correctly classifying the response (*i.e.* the Nat-Rnd class). Using these variables, we built a Neural Network to process the whole data by achieving a rate of correct classification of 94.36%.

The analysis of structure-related variables employed by the Neural Network is coherent with the descriptive statistical characteristics of variables distributions. In particular, alpha helix content (Figure 1a) and volume (Figure 2b) follow a bell-like distribution in the Rnd dataset. Conversely, the two structural features have a uniform-like distribution in the Nat ensemble. Two important insight emerged from this classification. First, it is possible to effectively identify the two different classes of proteins with a high degree of confidence. Second, a number of random proteins, 32 sequences, are erroneously classified as natural ones. This observation prompted an in-depth investigation of the structure of those random proteins misclassified as natural ones.

The fold analysis of random proteins misclassified by the ENNA algorithm showed that random polypeptides can adopt a great variety of conformations spanning from all-alpha to all-beta through complex mixed-folds. However the most representative fold we found was by far the all-alpha motif in approximately 32% of the proteins analyzed. Interestingly, the all beta fold was scarcely present accounting only for 3%. This result can be explained assuming that the average length (70 amino acids) of the random polypeptides does not suffice to construct a complete all-beta structure. On the other hand, one could advocate that the structural requirements for a beta-sheet formation (such as flatness, rigidity and pairing of beta strands far away from each other along the amino acid sequence) poses a number of constrains that cannot be met in completely random sequences, as already suggested in a previous study [16].

We also investigated whether misclassified random proteins assumed well-defined three-dimensional folds that show any resemblance to natural ones by assessing structural similarity using the DALI server [19] (http://ekhidna.biocenter.helsinki.fi/dali_server/).

**Figure 4:**
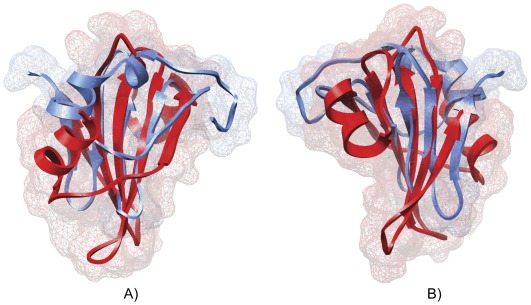
Superimposition of model A00927 and 1UUG. Schematic representation of the superposition of model A00927 (light blue) and the uracil-DNA glycosylase inhibitor protein (red) (PDB code 1UUG chain B). a) Front view, b) back view.

**Figure 5:**
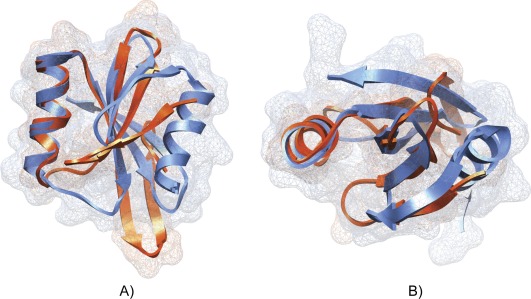
Superimposition of model A00084 and 2ZYZ. Schematic representation of the superposition of model A00084 (orange) and the *Pyrobaculum aerophilum* splicing endonuclease (light blue) (PDB code 2ZYZ chain A). a) front view, b) top view.

We identified 29 random proteins among the 32 misclassified by ENNA, which showed a general fold similarity, if not almost equal, to portion or sub-domains of natural proteins. In some cases the whole proteins were considerably similar to known natural proteins. The average RMSD obtained between the target protein and the query was characterized by a low value, equal to 3 Å. In addition, DALI ranked the results through the utilization of a Z-score which quantifies the “significant similarities” between two proteins. This value is an estimation of A) structural homology and B) sequence homology, and in general it strictly depends on the size of the query protein. As a reference point a Z-score value lower than 2 must be considered as a spurious result [19]. The obtained RMSD and Z-score values, in general good, should be perceived as exceptional if we consider the completely random nature of these proteins. In the entire misclassified subset, 22 proteins have a Z-score greater than 2; a value greater than 4 was found for the proteins A00927 and A00084. The protein A00927 is characterized by having the highest Z-score associated, equal to 4.4. The protein, is a large anti parallel beta-sheet, structurally related to the uracil-DNA glycosylase inhibitor protein (PDB code 1UUG chain B) with which it shares 9% sequence identity. The superposition (Figure 4a) reveals a high degree of structural homology on the central beta sheet spanning amino acids (W38-R68 of the random protein and I41-L84 of the natural one), good confidence was found also for a short alpha-helix present in the model and in the natural protein over amino acids (F16-L21 of the random protein and N3-G13 of the natural one) (Figure 4b). Due the diversity in the amino acid sequences is reasonable to assume that the synthetic protein A00927 does not show inhibitory activity. Similar results were obtained for the protein A00084 (Figure 5). The model appears to be structurally related to *Pyrobaculum aerophilum* splicing endonuclease (PDB code 2ZYZ chain A) (Z-score 4.3 and 5% of sequence identity). Also in this case DALI was able to identify a significant structural similarity. In general we can affirm that the biggest differences observed between our models and the natural proteins selected by DALI, could be attributed to the relative short length of the synthetic random polypeptides studied. As for the A00927 protein, the different amino acid sequence does not allow to conclude that protein A00084 has any endonuclease activitiy. Further investigations are necessary to clarify this aspect.

In order to corroborate these results we also verified that random proteins properly classified as non-natural did not show any significant structural similarity to natural ones. We analyzed 32 random proteins correctly classified as non-natural and analyzed their structural features using the same procedure employed for the misclassified subset. Properly classified random proteins display to lesser extent folds similar to natural proteins with an average Z-score of 1.7, significantly different from the average Z-score of random proteins misclassified as natural (Table 2).

**Table 2:** Overview of the structural analysis of classified random proteins.

Random proteins misclassified as natural				Random proteins correctly classified as non-natural			
Random Protein ID	Natural Protein Hit	RMSD (Å)	Z-score	Random Protein ID	Natural Protein Hit	RMSD (Å)	Z-score
00927	1ugg-B	2,5	4,4	00060	2fu2-A	2,9	3,5
00084	2zyz-A	2,9	4,3	00145	2zrr-A	3,6	3,5
00707	1w36-F	1,8	4,1	00070	1utx-A	2,7	2,8
00637	2it3-B	3,0	4,0	00080	1zgh-A	3,4	2,8
00905	21ah-A	3,8	4,0	00001	1bgw-A	3,9	2,6
00198	2ugi-B	2,9	3,8	00090	2zkm-X	2,7	2,6
00403	3kii-A	2,8	3,8	00095	2zlm-A	3,1	2,6
00135	3gtd-D	3,0	3,6	00130	2vlg-C	3,2	2,6
00229	3rk6-A	2,8	3,5	00055	2c41-B	3,3	2,5
00282	3qwh-A	3,4	3,5	00025	1sfu-A	4,2	2,3
00727	2waq-E	3,2	3,3	00030	1f7u-A	3,4	2,2
00585	3qnm-A	2,8	3,2	00045	3oee-N	3,9	2,2
00612	3icy-A	3,1	3,1	00115	2ld7-B	4,6	2,2
00892	3p1v-A	3,2	3,1	00050	3e26-A	2,9	2,1
00165	1n61-B	3,5	2,6	00065	2i5u-A	4,0	2,1
00301	1wlz-D	2,9	2,6	00015	3tl2-A	3,5	1,8
00746	1th5-A	2,8	2,6	00136	3pe3-A	3,2	1,5
00514	1qyz	3,0	2,4	00150	1vfg-B	3,2	1,4
00766	3ef6-A	3,1	2,4	00010	3so8-A	2,9	1,3
00296	3sbl-B	3,1	2,3	00040	2vhv2-B	3,2	1,1
00797	1kh0-B	3,0	2,2	00110	2jvg-A	3,9	1,0
00906	3kcn-B	3,0	2,1	00005	1lol-A	2,9	0,9
00398	3bxj-B	2,8	1,6	00035	3kgo-B	3,8	0,9
00083	1lsh-A	4,0	1,5	00085	3p9x-A	3,3	0,8
00687	3n2o-A	3,3	1,5	00125	2lc0-A	2,8	0,8
00391	1s7o-C	3,1	1,4	00155	3nnq-A	3,8	0,3
00708	2ooy-A	3,4	1,4	00020	no chain	n.a.	n.a.
00343	2jge-A	3,4	1,3	00100	no chain	n.a.	n.a.
00499	1zql-C	4,0	1,2	00105	no chain	n.a.	n.a.
00174	no chain	n.a.	n.a.	00120	no chain	n.a.	n.a.
00236	no chain	n.a.	n.a.	00140	no chain	n.a.	n.a.
00102	no chain	n.a.	n.a.	00075	no chain	n.a.	n.a.
	**Mean**	3,2	2,6		**Mean**	3,4	1,7
	**Dev Std**	0,3	0,9		**Dev Std**	0,5	0,7

Fold recognition of classified and misclassified random proteins. Both mean and variance were significantly different for Z-score and RMSD variables with a test significance level of 0.01.

All together, these results show that our algorithm is capable of effectively discriminate random protein from natural ones and that random proteins misclassified as natural by the ENNA algorithm display structural features strikingly similar to natural proteins.

## Discussion

Are extant proteins the exquisite result of natural selection or are they random sequences slightly edited by evolution? We address this question for the first time by comparing a set of 762 natural proteins and an equal number of completely random ones on the basis of their structural features. The first striking results is that random proteins do possess structural features comparable to those of natural proteins. However, the statistical indicators, such as mean and variance, of these structural-related variables significantly differ from those of naturally evolved polypeptides. In particular, random proteins show a narrower distribution with respect to natural ones. This can be regarded as a general feature of random amino acid polymers and it can be explained considering that random proteins represent statistical copolymers and therefore their structural features are centered around the mean with a variance equal to the one expected by the correspondent probability density function. Conversely, natural proteins display different mean and variance values, the latter being generally broader than the one of random proteins, due the result of the selective pressure that shaped natural protein structural features, leading to a deviation from expected values typical of statistical copolymers. This observation supports the idea that natural evolution has extensively refined proteins structural and chemo-physical properties to meet structural/functional requirements. In this regard, extant proteins cannot be regarded as simple edited random polypeptides, rather they clearly show the signature of selective pressure.

The differences are so remarkable that we were able to build a classification algorithm which effectively distinguishes natural proteins from random ones with an accuracy of 94.36%. In addition, random proteins misclassified as natural ones are characterized by structural similarity to natural proteins. In particular, misclassified random proteins exhibit a significant fold similarity to portions or subdomains of extant proteins at atomic resolution.

These results support the idea that random polypeptides do possess intrinsic structural features that render them particularly suitable for natural selection. In particular, secondary structure elements and well-defined folds are readily detected among completely random proteins. These intrinsic structural characteristics are then systematically tuned and shaped by the action of evolutionary optimization. This scenario is consistent with experimental results which show that compact and thermodynamically stable proteins can be easily found screening small libraries of completely random sequences by phage display [20] and functional proteins can be selected in vitro [21], [22] or in vivo [23] from random sequences libraries in relatively few evolutionary cycles. A similar scenario has been proposed also for other biopolymers such as single-stranded RNA [24], [25].

Our results suggest that random proteins are significantly different from extant ones, yet they display inherent conformational order which derives from chemico-physical constrains rather than from natural selection. This intrinsic order represents a “free-ticket” to start the adaptation process to specific functions and environments.

## Materials and Methods

### Random Protein Sequence Generation

Random sequences employed for this study were generated using the RandomBlast algorithm described elsewhere [26]. The RandomBlast algorithm consists of two main modules: a pseudo random sequence generation module and a Blast software interface module. The first module uses the Mersenne Twister 1973 pseudo-random number generation algorithm [27] to generate pseudo-random numbers between 0 and 19. To each amino acid is assigned a fixed number and single amino acids are then concatenated to reach the sequence length of 70 amino acids used in this work. Each generated sequence is then given in input to the second RandomBlast module, an interface to the Blast blastall program which invokes the following command:

blastall -m 8 -p blastp -d database -b 1;

where database in our case stands for the NR database [28], [29], and the parameters –m 8 and –b 1 indicate the alignment format (tabular form) and the number of sequences to be returned (just the first hit), respectively. In our case we regard as valid only the protein sequences that do not display significant similarity to any natural protein present in the database. In other words, contrary to the normal Blast usage, Randomblast consider as valid only completely random sequences. The sequence length of 70 amino acids was chosen as a good compromise between the computational requirements and the scientific investigation.

### In silico Random Proteins Structure Prediction and Fold Analysis

The three-dimensional model structures of random proteins were predicted using Rosetta Abinitio, an ab initio protein structure prediction software based on the assumption that in a polypeptide chain local interactions bias the conformation of sequence fragments, while global interactions determine the three-dimensional structure with minimal energy [30]. For each sequence 20.000 decoys were predicted. The decoys were clustered using the Rosetta clustering integrated module. Only the first model proposed for each sequence was taken into consideration. Detailed fold analysis was conducted only for the 32 proteins misclassified by ENNA. The DALI protein structure database searching web server was used [31]. The output of DALI is characterized by a long list of putative results, ranked by RMSD and Z-score. Only the best candidate ranked by Z-score was considered.

### Statistical Analysis of the Data

The statistical analyses were performed using the R-project free software environment for statistical computing and graphics (www.r-project.org), version R 2.10.1 GUI 1.31 Leopard build 32-bit. We performed the explorative analyses by using the standard *stats* package. We tested the Gaussianity assumption for the variables by calculating the Shapiro test. We compared the mean and the variance of each variable distributions in the two classes of the proteins by using non parametric tests, namely the Wilkoxon test for the mean and the Fligner-Killen test for the variance.

### The Evolutionary Neural Network Algorithm

ENNA is built in R environment by combining functions of the package *RWeka* [32], version 0.4–1, and of the package *genalg*, version 0.1.1. More specifically, we generated a first random population of networks with the topology of a 2-hidden layers neural networks by using the function make_Weka_classifier [32] fixing a MultilayerPerceptron interface function. The nodes in the network are sigmoid and learning rate for the parameters updates was fixed to 0.3. The population was formally described as a set of sequences with dichotomic variables (each sequence is a vector of zeros - ones values), representing the input of each network. Each element of the sequence described the presence or the absence of a particular structural component of a protein in terms of the variables considered in the analysis. The topology of these networks, involving different variable compositions, was selected in a random way (first generation of networks), and the response of each network was derived with a two classes structure: natural and random proteins. We then built a genetic algorithm by using the function rbga.bin{genalg} to evolve the population of networks in a number of generations to identify a precise classification rule. We evaluated the response of each network deriving a net misclassification rate by 10-fold cross validation procedure: the sequences with smaller values were identified as the more promising solutions. Then we applied to the network population the classical genetic operators, such as natural selection, crossover and mutation, in order to achieve the next generation of promising sequences. In particular we adopted a roulette wheel selection method, where the probability that each sequence (representing a specific neural network topology) to be selected is proportional to its fitness score, namely its misclassification rate. The next population was achieved by applying a single point crossover method and a mutation operator. In particular, the crossover method was used in a way that a point of exchange was randomly set in the two individual sequences. Then, the mutation operator was applied changing each element of the sequence with a probability fixed to 0.01. Each population was composed of 30 sequences which evolve across 10 generations.
